# GhXB38D represses cotton fibre elongation through ubiquitination of ethylene biosynthesis enzymes GhACS4 and GhACO1

**DOI:** 10.1111/pbi.14138

**Published:** 2023-08-19

**Authors:** Qingwei Song, Wanting Gao, Chuanhui Du, Wenjie Sun, Jin Wang, Kaijing Zuo

**Affiliations:** ^1^ Single Cell Research Center, School of Agriculture and Biology Shanghai Jiao Tong University Shanghai China; ^2^ Biotechnology Research Institute Chinese Academy of Agricultural Sciences Beijing China

**Keywords:** cotton, fibre elongation, ethylene, 1‐aminocyclopropane‐1‐carboxylate oxidase (aco), 1‐aminocyclopropane‐1‐carboxylate synthase (acs), ubiquitination

## Abstract

Ethylene plays an essential role in the development of cotton fibres. Ethylene biosynthesis in plants is elaborately regulated by the activities of key enzymes, 1‐aminocyclopropane‐1‐carboxylate oxidase (ACO) and 1‐aminocyclopropane‐1‐carboxylate synthase (ACS); however, the potential mechanism of post‐translational modification of ACO and ACS to control ethylene synthesis in cotton fibres remains unclear. Here, we identify an E3 ubiquitin ligase, GhXB38D, that regulates ethylene biosynthesis during fibre elongation in cotton. *GhXB38D* gene is highly expressed in cotton fibres during the rapid elongation stage. Suppressing *GhXB38D* expression in cotton significantly enhanced fibre elongation and length, accompanied by the up‐regulation of genes associated with ethylene signalling and fibre elongation. We demonstrated that GhXB38D interacts with the ethylene biosynthesis enzymes GhACS4 and GhACO1 in elongating fibres and specifically mediates their ubiquitination and degradation. The inhibition of *GhXB38D* gene expression increased the stability of GhACS4 and GhACO1 proteins in cotton fibres and ovules, resulting in an elevated concentration of ethylene. Our findings highlight the role of GhXB38D as a regulator of ethylene synthesis by ubiquitinating ACS4 and ACO1 proteins and modulating their stability. *GhXB38D* acts as a negative regulator of fibre elongation and serves as a potential target for enhancing cotton fibre yield and quality through gene editing strategy.

## Introduction

Cotton is the main source of renewable natural fibres extensively used in fabric production within the textile industry. Fibre length is an important index of cotton fibre quality that affects the finesse and rank of fabrics (Sun *et al*., 2019; Zhang *et al*., 2010). The development of cotton fibre typically spans approximately 50 days, from the initiation of fibre cells to the formation of mature fibres (Basra and Malik, 1984). This process can be divided into four stages: fibre initiation, fibre elongation, secondary wall thickness and mature fibre development. Among these stages, fibre elongating, which lasts approximately from 1 day post‐anthesis (DPA) to 20 DPA, significantly influences the final length of the mature cotton fibre (Haigler *et al*., 2012).

Plant hormones, including ethylene, brassinosteroids (BRs) and gibberellins (GAs), play essential roles in the elongation and expansion of cotton fibres (Fang *et al*., 2017; Luo *et al*., 2007; Qin *et al*., 2007; Shi *et al*., 2006). Transcriptomic and metabolic pathway analyses have revealed that the ethylene biosynthesis pathway is particularly significant in enhancing fibre elongation (Fang *et al*., 2017; Qin *et al*., 2007). Application of the ethylene synthesis precursor 1‐aminocyclopropane‐1‐carboxylic acid (ACC) in ovule *in vitro* culture significantly promotes fibre elongation, whereas addition of the ethylene synthesis inhibitor L‐(2‐aminoethoxyvinyl)‐glycine (AVG) to the culture medium retards fibre elongation (Qin *et al*., 2007; Shi *et al*., 2006). Ethylene directly up‐regulates the expression of genes involved in fibre cell cytoskeleton construction (e.g., *ACTIN1* gene), secondary cell wall synthesis (e.g., *CESA1*) and fibre cell elongation (e.g., *EXPA1*) (Pang *et al*., 2010; Shi *et al*., 2006). Application of C24:0 fatty acids to ovules cultured *in vitro* resulted in a significant increase in ethylene production and ACO transcript levels during fibre elongation (Qin *et al*., 2007; Wang *et al*., 2011). Furthermore, ethylene interacts with the signalling pathways of BRs and GAs, exerting hormonal effects on fibre cell elongation (Qin *et al*., 2007; Wang *et al*., 2002). Ethylene treatment can counteract the inhibition of fibre growth after application of the BR inhibitor BRZ, whereas BR does not recover the fibre shortening induced by AVG application. These observations suggest that the stimulatory effect of BR may occur downstream of the ethylene signalling pathway (Shi *et al*., 2006). Exogenous application of GA3 significantly up‐regulates the expression of most 3‐ketoacyl‐CoA synthase (*KCS*) genes controlled by ethylene signalling, promoting fibre length and further highlighting the crucial role of ethylene in regulating fibre elongation (Xiao *et al*., 2016; Zhang *et al*., 2018).

In plants, the biosynthesis of ethylene from S‐adenosylmethionine (SAM) involves two steps: SAM is firstly converted to ACC by ACC synthases (ACSs); ACC is further catalysed by ACC oxidases (ACOs) to produce ethylene (Ververidis and John, 1991; Yang and Hoffman, 1984). ACSs and ACOs, as rate‐limiting enzymes, are major targets for the regulation of ethylene production at the transcriptional and post‐translational levels during plant development (Carvalho *et al*., 2012; Han *et al*., 2010; Joo *et al*., 2008; Lyzenga *et al*., 2012). For instance, the stability of ACS and ACO proteins in *Arabidopsis* is controlled by proteasome‐dependent protein degradation (Hao *et al*., 2021; Jin *et al*., 2017; Merchante *et al*., 2013). At the post‐translational level, the stability of type 2 and type 3 ACS proteins relies on the activity of RING‐type E3 ligases (Stone *et al*., 2005). In addition, AtACS7 can be degraded by the ubiquitin‐26S proteasome pathway through its interaction with XBAT32, a RING‐type E3 ligase involved in ethylene homoeostasis (Lyzenga *et al*., 2012). Notably, XBAT32 is required for the degradation of AtACS4 and AtACS7 in *Arabidopsis* and interacts with and ubiquitinates AtACS4 and AtACS7 *in vitro* (Nodzon *et al*., 2004; Prasad *et al*., 2010). *Arabidopsis* XBAT32 mutants produce excessive ethylene and display a range of phenotypes associated with ethylene, such as the triple response in the darkness. These findings suggest that ubiquitin‐mediated protein degradation patterns allow *Arabidopsis* plants to respond more precisely to developmental and stress signals (Jin *et al*., 2017; Konishi and Yanagisawa, 2008; Stone and Callis, 2007). Considering the evolutionary conservation of the components in the ethylene synthesis pathway across plant species (Yang *et al*., 2015), we questioned whether negative regulatory mechanisms of ethylene synthesis exist in the control of cotton fibre elongation.

Previous reports have demonstrated that transcripts of *GhACO1~3* rapidly accumulate in elongating fibres following fibre initiation (Fang *et al*., 2017; Qin *et al*., 2007; Shi *et al*., 2006). The expressions of *GhACO*s and *GhACS*s are activated by Ca^2+^‐dependent protein kinase 1 (CDPK1), which helps maintain ethylene concentrations in fibres and ovules to sustain fibre cell expansion (Wang *et al*., 2011), supporting the importance of the high expression of ethylene biosynthetic gene transcripts for fibre elongation. However, high expression of the ethylene biosynthesis genes *ACO1* and *ACO3* impairs fibre elongation in diploid cotton species (Li *et al*., 2014). Furthermore, there is no positive correlation between ethylene content, transcript levels of ethylene synthesis genes and mature fibre length in cultivars (Fang *et al*., 2017; Jiang *et al*., 2022). These observations suggest that the regulation of ethylene production is more elaborated than the mere control of the transcript levels of ethylene synthesis genes (Guo and Ecker, 2003; Han *et al*., 2010; Kendrick and Chang, 2008; Koyama, 2014; Merchante *et al*., 2013). Precise control of ethylene synthesis at both transcriptional and post‐translational levels is necessary to support cotton fibre elongation.

Although the impact of ethylene in promoting cotton fibre elongation has been characterized, the mechanisms governing ethylene synthesis in cotton fibres remain largely unknown (Huang *et al*., 2021; Zhu *et al*., 2021). Previous studies have indicated that several *XB3* genes encoding E3 ubiquitin ligases in cotton are highly expressed during fibre elongation and are likely involved in the regulation of ethylene synthesis and signal transduction (Ge *et al*., 2021; Sun *et al*., 2019). To elucidate the post‐translational regulatory mechanisms of ethylene biosynthesis in cotton fibres, we conducted a comprehensive analysis of reported cotton transcriptomic and proteomic databases to identify candidate genes within the *XB3* gene family. In this study, we characterized the function of *GhXB38D* and confirmed that GhXB38D protein can mediate the ubiquitination and degradation of GhACS4 and GhACO1 proteins. In *GhXB38D* RNAi lines, the stability of GhACS4 and GhACO1 proteins was improved, leading to increased ethylene content and fibre elongation in cotton. In conclusion, our results demonstrate that *GhXB38D* negatively regulates fibre development by influencing ethylene biosynthesis.

## Results

### *GhXB38D* is functionally associated with fibre elongation

The *XB3* gene family in plants encode a kind of E3 ubiquitin ligases with ankyrin‐repeat (ANK) and RING domains, which play crucial roles in plant growth, development and stress responses (Carvalho *et al*., 2012; Deshaies and Joazeiro, 2009; Huang *et al*., 2006; Prasad *et al*., 2010). To explore the roles of the *XB3* gene family in cotton fibre elongation, we performed HMMER alignment (www.hmmer.org/), searching for *XB3* gene families in the cotton genus (*Gossypium*), including the tetraploid cotton *G. hirsutum* and two diploid species *G. arboreum* (A2) and *G. raimondii* (D5). After exclusion of repetitive sequences and the encoded proteins lacking the RING (PF12796) or ANK domain (PF13920), we identified 32 unique *XB3* genes from different cotton genomes (Table S1). This set included 16 *XB3* genes from *G. hirsutum*, 9 *XB3* genes from *G. arboretum* and 7 *XB3* genes from *G. raimondii*. To maintain consistency, we renamed the cotton *XB3* genes based on their homologues in the rice genome (*Oryza sativa*) (Nodzon *et al*., 2004); that is, *XB3* genes from *G. hirsutum, G. arboretum* and *G. raimondii* were renamed *GhXB3s, GaXB3s* and *GrXB3s*, respectively.

To investigate the evolutionary relationship between GhXB3s and orthologs from *Arabidopsis thaliana, Oryza sativa, G. arboreum* and *G. raimondii*, we constructed a neighbour‐joining (NJ) phylogenetic tree based on 45 XB3 proteins (Figure S1a). The XB3s were classified into three major clades (I–III). The largest clade I included 16 cotton XB3s numbered 31, 36, 37 and 38 (GaXB31, 36, 37 and 38; GrXB31, 36, 37 and 38; GhXB31, 36, 37, 38A and D). Clade II contained 25 XB3 proteins, 11 of which were cotton XB3s numbered 32, 33 and 34 (GaXB32, 33; GrXB32, 33, 34; GhXB32, 33, 34 A and D). Clade III consisted of five cotton XB35s (GaXB35, GrXB35, 35–1; GhXB35A, and 35D) (Figure S1a). The number of XB3 family members in *G. hirsutum* was the sum of the two diploid cotton species, indicating that XB3 family genes are evolutionarily conserved in the cotton lineage.

**Figure 1:**
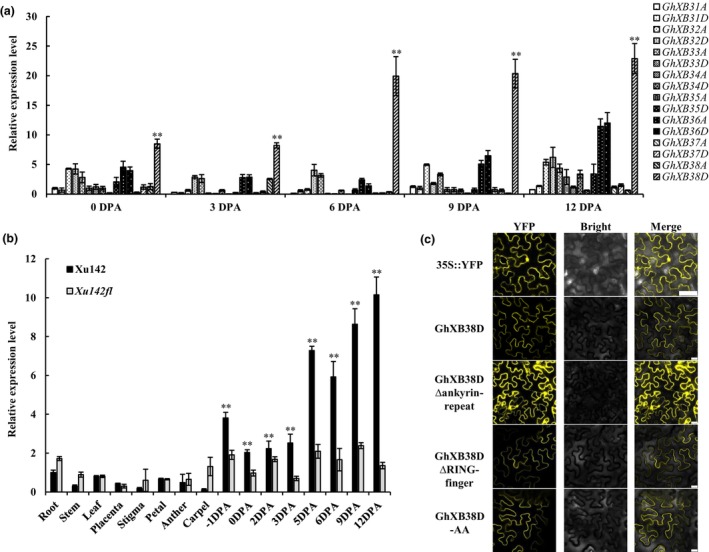
*GhXB38D* is highly expressed in cotton elongating fibres. (a) Expression profile of the *GhXB3* gene family during cotton fibre development (from 0 to 12 DPA) in Xu142. Gene expression data were obtained by quantitative real‐time PCR with three independent replicates. Error bars indicate the standard errors of independent biological replicates (*P < 0.05; **P < 0.01 by Student's *t*‐test). (b) Comparative expression of *GhXB38D* in different tissues (root, stem, leaf, placenta, stigma, petal, anther and carpel) and developing cotton fibres (from −1 to 12 DPA) of Xu142 and its fibreless mutant *Xu142fl*. Gene expression data were obtained by quantitative real‐time PCR with three independent replicates. Error bars indicate the standard error of independent biological replicates (*P < 0.05; **P < 0.01, by Student's *t*‐test). (c) Subcellular localization of the GhXB38D protein. Fluorescence of 35S::YFP, GhXB38D‐YFP, GhXB38D Δankyrin‐repeat‐YFP, GhXB38D ΔRING‐finger‐YFP and GhXB38D‐AA‐YFP fusion proteins in epidermal cells of tobacco (*Nicotiana benthamiana*) leaves. Bars = 50 μm.

To assess the involvement of *GhXB3* genes in cotton fibre elongation, we analysed the expression profiles of all the *GhXB3* family genes at different development stages of fibres. Quantitative real‐time PCR (qRT‐PCR) analysis revealed that *GhXB38D* transcripts gradually increased from 0 DPA, reaching their peak level at 12 DPA, significantly exceeding the expression of other *GhXB3* genes during corresponding developmental stages (Figure 1a). We further compared *GhXB38D* gene expression in different tissues and fibre development stages between Xu142 and its fibreless mutant *Xu142fl*. *GhXB38D* expression was low in different tissues, except in rapidly elongating fibres (5–12 DPA, Figure 1b). *GhXB38D* transcripts in the elongating fibres of Xu142 were approximately 1.5‐ to 3.5‐fold higher compared with those in *Xu142fl* (Figure 1b). RNA *in situ* hybridization assays further indicated that *GhXB38D* was specifically expressed in developing fibres, and in the outer and inner seed coats of ovules at 3 DPA (Figure S2). Collectively, these findings suggest a close association between the function of *GhXB38D* and cotton fibre elongation.

### GhXB38D is localized in the plasma membrane and nucleus

Protein sequence alignment revealed that GhXB38D shares 51% identity with AtXBAT31, which is involved in protein ubiquitination and hypocotyl growth in *Arabidopsis* (Figure S1c, Zhang *et al*., 2021). A conserved domain search conducted online (http://www.ncbi.nlm.nih.gov/blast) revealed that *GhXB38D* gene encodes a typical RING E3‐ligase with an ANK domain at the N‐terminal (54–249 aa) and a C3HC4‐type RING finger motif at the C‐terminal (250–371 aa) (Figure S1b,c).

To investigate the subcellular localization of GhXB38D *in vivo* and the impact of domain deletion on subcellular protein location, GhXB38D and its domain‐deleted variants (GhXB38D Δankyrin‐repeat and GhXB38D ΔRING finger) were fused with YFP and transiently expressed in the epidermal cells of tobacco leaves. Fluorescence signals of GhXB38D‐YFP were observed in both the cell membrane and nucleus of tobacco epidermal cells. In contrast, the YFP signal of the protein lacking the ankyrin‐repeat domain (GhXB38D Δankyrin‐repeat) accumulated in the cytoplasm and nucleus, and the YFP signal of the GhXB38D protein lacking the RING finger domain was detected exclusively at the cell membrane (Figure 1c). These findings suggest that the ankyrin‐repeat domain is necessary for membrane targeting of the GhXB38D protein. Next, we introduced the mutations in two zinc coordination residues (Cys‐338 and His‐340), which are essential for E3 ligase function by replacing them with Ala (Figure S1c). We then fused the mutant protein (GhXB38D‐AA) with YFP and analysed its subcellular localization. The fluorescent signals demonstrated that GhXB38D‐AA‐YFP and GhXB38D‐YFP were equally localized at the cell membrane and nucleus, indicating that mutations in key residues of the GhXB38D protein do not affect its subcellular localization (Figure 1c).

### Inhibition of *GhXB38D* expression promotes cotton fibre elongation

**Figure 2:**
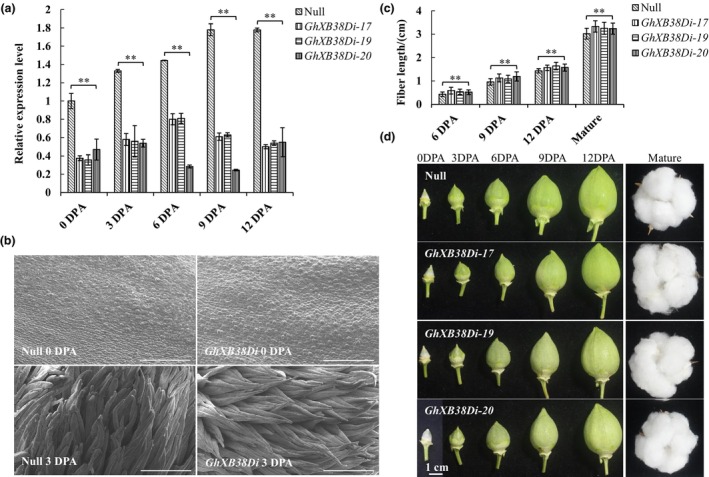
Suppression of *GhXB38D* expression in cotton promotes fibre elongation. (a) Relative expression level of *GhXB38D* in null plants and *GhXB38D* RNAi lines (*GhXB38Di‐17, 19* and *20*) during fibre development (from 0 to 12 DPA). Error bars represent ± SE of three biological replicates (*P < 0.05; **P < 0.01, by Student's *t*‐test). (b) Scanning electron microscope (SEM) images show the ovule surfaces of null plants and *GhXB38Di* lines at 0 and 3 DPA. SEM images show the epidermal cells in the centre of the ovule. Bars = 100 μm. (c) Average fibre length of null plants and *GhXB38D* RNAi lines (*GhXB38Di‐17, 19* and *20*). More than 100 seeds per line were used for statistical analysis. Error bars represent ±SE of three biological replicates (*P < 0.05; **P < 0.01, by Student's *t*‐test). (d) Comparison of cotton bolls from null plants and *GhXB38Di* lines (*GhXB38Di‐17, 19* and *20*) during fibre development (from 0 DPA to 12 DPA and at maturity). Bars = 1 cm.

To investigate the function of *GhXB38D* in fibre development, we employed Agrobacteria‐mediated transformation to generate *GhXB38D* RNA interference (RNAi) plants using *G. hirsutum* var. Jimian 14 as the host plant. PCR analysis confirmed the successful integration of the *GhXB38D* RNAi cassette into the cotton genome (Figure S3), and qRT‐PCR analysis revealed that *GhXB38D* RNAi lines exhibited at least 44.5% inhibition of *GhXB38D* expression during fibre elongation (Figure 2a). Three positive T_0_ plants were randomly selected to generate independent homozygous T_4_ lines (*GhXB38Di‐17, GhXB38Di‐19* and *GhXB38Di‐20*) for subsequent experiments. Negative segregates lacking the *GhXB38D* RNAi construct from the T_1_ lines were self‐crossed and used as null controls. The *GhXB38Di* T_4_ lines and null plants were grown in the field for two consecutive years (2020 and 2021) to analyse the effects of *GhXB38D* expression on fibre development, fibre quality and yield components.

Cryoelectron microscopic analysis revealed that the number of fibre initiation cells in *GhXB38Di* lines exhibited no significant differences from that in the null plants (Figure 2b). However, after fibre initiation, *GhXB38Di* lines exhibited significantly faster fibre elongation compared to null plants at 3 DPA (Figures 2b and S4). Fibre length comparisons demonstrated that the *GhXB38Di* lines were 19.5%~34.5%, 13.5%~24.5%, 9.1%~14.3% and 7.3%~10.4% longer than the null plants at 6, 9 and 12 DPA and mature, respectively (Figure 2c). In 2020 and 2021, the lint index of the *GhXB38Di* lines increased by 15.7%~18.8% and 20.4%~28.8%, and the lint percentage also increased by 9.7%~11.3% and 13.2%~14.6% compared to the null plants (Table 1). Furthermore, fibre quality testing using a Premier HFT 9000 demonstrated that the average length of mature fibres in the upper half of the *GhXB38Di* lines was 5.9%~10.3% longer than that of the null plants (Table 2). The fibre strength and elongation rate of *GhXB38Di* lines increased by 6.6%~7.6% and 7.0%~13.4%, respectively (Table 2). Overall, these results indicate that the inhibition of *GhXB38D* expression increases the elongation rate and length of cotton fibre cells without altering the phenotype of plant vegetative growth (Figure S5), suggesting that *GhXB38D* negatively regulates fibre elongation.

**Table 1:** The lint index, seed index and lint percentage of the *GhXB38D*‐RNAi transgenic cotton plants and transgene‐negative null plants

	Lint index (g)	Seed index (g)	Lint percentage (%)
2020			
Null	6.56 ± 0.71	9.74 ± 0.55	40.29 ± 1.14
*GhXB38Di‐17*	7.67 ± 0.50*	9.65 ± 0.39	44.27 ± 0.60*
*GhXB38Di‐19*	7.79 ± 0.25**	9.83 ± 0.16	44.21 ± 0.64*
*GhXB38Di‐20*	7.59 ± 0.20**	9.37 ± 0.83	44.86 ± 1.51*
2021			
Null	6.81 ± 0.51	10.12 ± 0.61	40.22 ± 0.75
*GhXB38Di‐17*	8.20 ± 0.30*	9.48 ± 0.66	46.41 ± 1.53**
*GhXB38Di‐19*	8.26 ± 0.34*	9.87 ± 0.37	45.54 ± 0.36**
*GhXB38Di‐20*	8.77 ± 0.54*	10.25 ± 0.56	46.09 ± 0.80**

Significant differences among the different lines according to Student's *t*‐test analysis: *P < 0.05; **P < 0.01. Values were the mean ± SD of assays for three repetitions of each line.

**Table 2:** Comparison of fibre quality between GhXB38D‐RNAi cotton plants and transgene‐negative null plants

	Average length of upper half (mm)	Strength (cN•tex‐1)	Micronaire	Uniformity index (%)	Elongation rate (%)
Null	27 ± 0.71	29.17 ± 0.17	5 ± 0.71	85.4 ± 0.33	5.73 ± 0.12
*GhXB38Di‐17*	29.77 ± 0.50*	31.4 ± 0.57**	5.33 ± 0.12	85.9 ± 0.54	6.5 ± 0.29*
*GhXB38Di‐19*	28.6 ± 0.36*	31.4 ± 0.86*	5.43 ± 0.09	85.47 ± 0.52	6.17 ± 0.12*
*GhXB38Di‐20*	28.73 ± 0.21*	31.1 ± 0.70*	5.43 ± 0.05	86.9 ± 0.43	6.13 ± 0.12*

Significant differences among the different lines according to Student's *t*‐test analysis: *P < 0.05; **P < 0.01. Values were the mean ± SD of assays for three repetitions of each line.

### GhXB38D interacts with the ethylene biosynthesis enzymes GhACS4 and GhACO1

**Figure 3:**
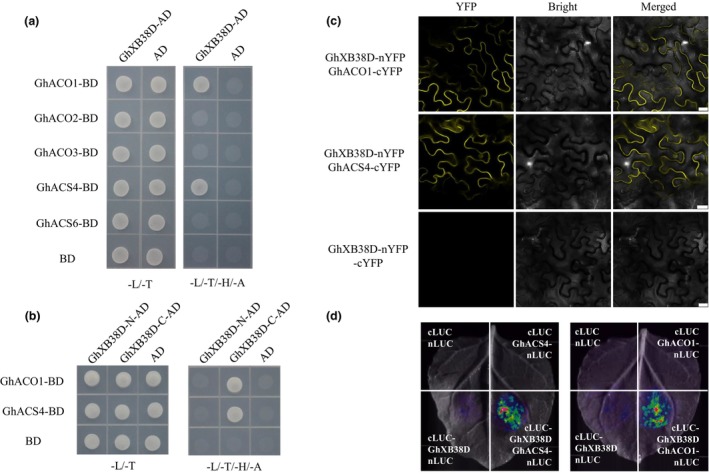
GhXB38D physically interacts with GhACO1 and GhACS4 *in vivo* and *in vitro*. (a) Interaction of GhXB38D with GhACS4 and GhACO1 was demonstrated by yeast two‐hybrid assay. AD, Gal4 activation domain fusion; BD, Gal4 DNA‐binding domain fusion. Negative control: AD‐GhXB38D with BD; AD with BD‐GhACO1; AD with BD‐GhACS4; AD with BD. Yeast growth was confirmed on ‐L‐T medium (lacking leucine and tryptophan) and any interaction was monitored on ‐L‐T‐H‐A medium (lacking leucine, tryptophan, histidine and adenine). (b) The interaction of GhACO1 and GhACS4 with the GhXB38D domain was verified by yeast two‐hybrid assay. (c) GhXB38D interacts with GhACO1 and GhACS4 in tobacco leaf epidermal cells using BIFC assay. Scale bar = 50 μM. (d) GhXB38D interacted with GhACO1 and GhACS4 in tobacco leaf epidermal cells using a split luciferase assay. GhXB38D, GhACO1 and GhACS4 were fused to the N‐terminus (nLUC) and C‐terminus (cLUC) of firefly luciferase, respectively. The empty vector was used as a control. The different combinations of constructs were infiltrated into tobacco leaves and observed for chemiluminescence.

XBAT32, a protein involved in regulating lateral root development in *Arabidopsis*, exerts its function through the ubiquitination of ethylene biosynthetic proteins AtACS4 and AtACS7 (Lyzenga *et al*., 2012; Prasad *et al*., 2010). Previous studies have suggested that two *ACS* genes (*GhACS4* and *GhACS6*) and three *ACO* genes (*GhACO1~3*) in the *ACS* and *ACO* gene families of upland cotton may play a role in ethylene synthesis during fibre development (Li *et al*., 2022; Shi *et al*., 2006). Transcriptome data and qRT‐PCR results confirmed the strong expression of *GhACS4, GhACS6, ACO1, ACO2* and *GhXB38D* during the rapid elongation phase of fibres, whereas *ACO3* gene was highly expressed during fibre initiation and in vegetative tissues (Figures 1b and S6, Table S2). Therefore, we employed a yeast two‐hybrid system to investigate the interactions between GhXB38D and GhACSs, or GhACOs. As shown in Figure 3a, yeast cells co‐transformed with GhXB38D and either GhACS4 or GhACO1 grew normally on SD medium without leucine, tryptophan, histidine or adenine, indicating that GhXB38D interacts with GhACS4 and GhACO1 *in vitro*. Additionally, we demonstrated that this interaction occurs through the RING finger domain rather than through the ankyrin‐repeat domain (Figure 3b). To confirm these interactions *in vivo*, BiFC assays were conducted on epidermal cells of *N. benthamiana* leaves. Strong fluorescent signals of YFP were detected throughout the epidermal cells expressing GhXB38D‐YN with GhACS4‐YC or GhACO1‐YC, while co‐expression of GhXB38D with the control protein failed to produce YFP fluorescent signals (Figure 3c). Furthermore, luciferase complementary imaging (LCI) assays performed in the epidermal cells of the tobacco leaves confirmed these interactions (Figure 3d). Luciferase signal screening showed that cLUC‐GhXB38D co‐transformed with GhACS4‐nLUC or GhACO1‐nLUC produced stronger fluorescent signals in epidermal cells than in controls (Figure 3d). Collectively, these data suggest that GhACS4 and GhACO1 interact with GhXB38D.

### GhXB38D mediates the ubiquitination and degradation of GhACS4 and GhACO1 proteins in cotton

**Figure 4:**
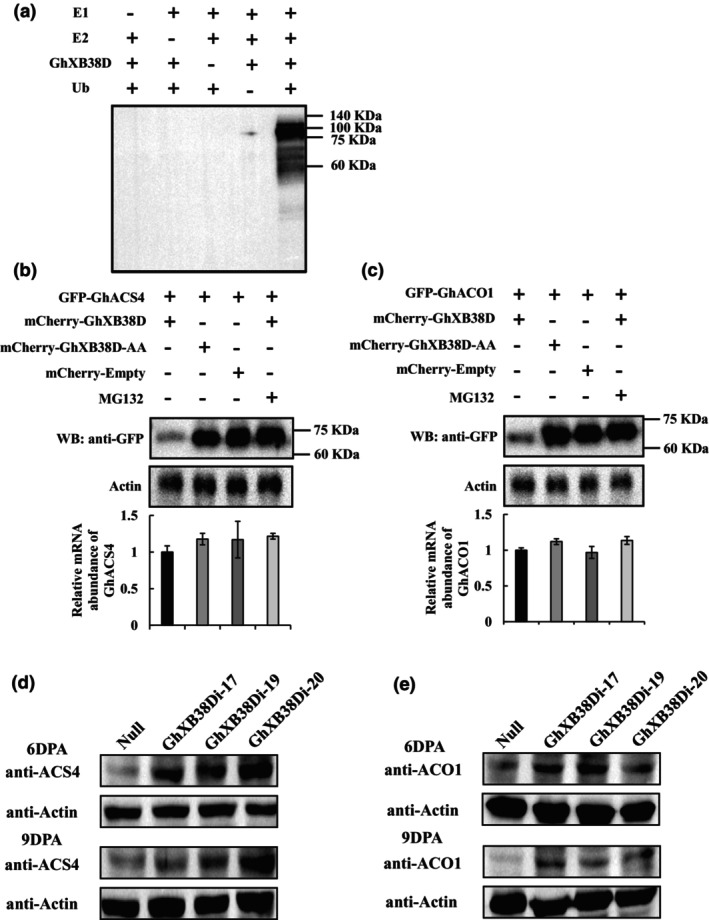
GhXB38D mediates the degradation of GhACS4 and GhACO1 proteins through *in vitro* and *in vivo* ubiquitination assays. (a) In vitro E3 ubiquitin ligase activity of the GhXB38D. MBP‐labelled recombinant GhXB38D protein was incubated for 2 h at 28 °C in the presence or absence of *Arabidopsis* E1 (UBA2), E2 (UBC10) or ubiquitin. Samples were analysed by 10% SDS‐PAGE, and GhXB38D protein was detected by Western blotting with anti‐ubiquitin antibody. Molecular weight (kDa) is indicated on the right side of the gel. (b, c) In vivo ubiquitination of GhACS4 and GhACO1 by GhXB38D. GhACS4 and GhACO1 fused to GFP were expressed alone or coexpressed with GhXB38D or GhXB38D‐AA in tobacco leaves in the presence or absence of MG132. Protein extracts were analysed using anti‐GFP antibodies. Actin was used as a loading control. Molecular weight (kDa) is indicated on the right side of the gel. The relative mRNA abundance of *GhACS4* and *GhACO1* in each sample is shown at the bottom of the image. Each value represents the mean ± SE of three biological replicates. (d, e) Protein levels of GhACO1 (d) and GhACS4 (e) in ovules and fibres of null plants and *GhXB38Di* lines at different developmental stages (6DPA and 9DPA) were detected by Western blotting. The proteins of GhACS4 and GhACO1 were detected with synthetic specific antibodies anti‐GhACS4 and anti‐GhACO1, respectively. Actin was used as the loading control.

To investigate whether GhACS4 or GhACO1 are degraded through GhXB38D‐mediated ubiquitination by the 26S proteasome, we performed both *in vitro* and *in vivo* ubiquitination assays. Several RING finger‐based E3 proteins can catalyse auto‐ubiquitination in an E2‐dependent manner (Komander and Rape, 2012; Lorick *et al*., 1999). To assess the E3 activity of GhXB38D, we examined its ability to mediate auto‐ubiquitination *in vitro*. The recombinant protein MBP‐GhXB38D was incubated in the presence or absence of *Arabidopsis* proteins E1 (UBA2), E2 (UBC10) and ubiquitin (Ub). Ubiquitination products with higher molecular weights were observed only in the presence of E1, E2 and Ub, whereas no ubiquitination occurred in the absence of either condition (Figure 4a), supporting the hypothesis that GhXB38D has E3 ligase and auto‐ubiquitination activities.

To examine whether GhXB38D mediates the degradation of GhACS4 and GhACO1, we assessed the stability of GhACS4 and GhACO1 in tobacco and cotton fibre cells using Western blotting. As depicted in Figure 4b,c, the protein levels of GhACS4 or GhACO1 decreased significantly in the presence of GhXB38D, whereas GhACS4 and GhACO1 remained relatively stable in the presence of GhXB38D‐AA or in the absence of GhXB38D. Additionally, the protein degradation of GhACS4 or GhACO1 mediated by GhXB38D was inhibited by MG132 (Figure 4b,c). In the BiFC assay, the fluorescence intensity of tobacco cells co‐expressing GhXB38D‐AA, GhACS4 or GhACO1 was significantly increased due to mutations in the two amino acids required for E3 ligase activity (Figure S7). Furthermore, the fluorescence intensity of GhACS4 or GhACO1 interacting with GhXB38D significantly increased following treatment with MG132 (Figure S7). We further examined the protein levels of GhACS4 and GhACO1 in ovules and fibres of null plants and *GhXB38Di* lines using anti‐GhACS4 and anti‐GhACO1 antibodies, respectively. Western blotting revealed significantly higher GhACS4 and GhACO1 protein levels in the ovules and fibres of the *GhXB38Di* lines (6DPA and 9DPA) compared to the null plants (Figure 4d,e). These findings strongly suggest that GhXB38D mediates the degradation of GhACS4 and GhACO1 via a ubiquitin‐dependent pathway.

### Inhibition of *GhXB38D* in cotton up‐regulates the expression of fibre development‐related and ethylene‐responsive genes

**Figure 5:**
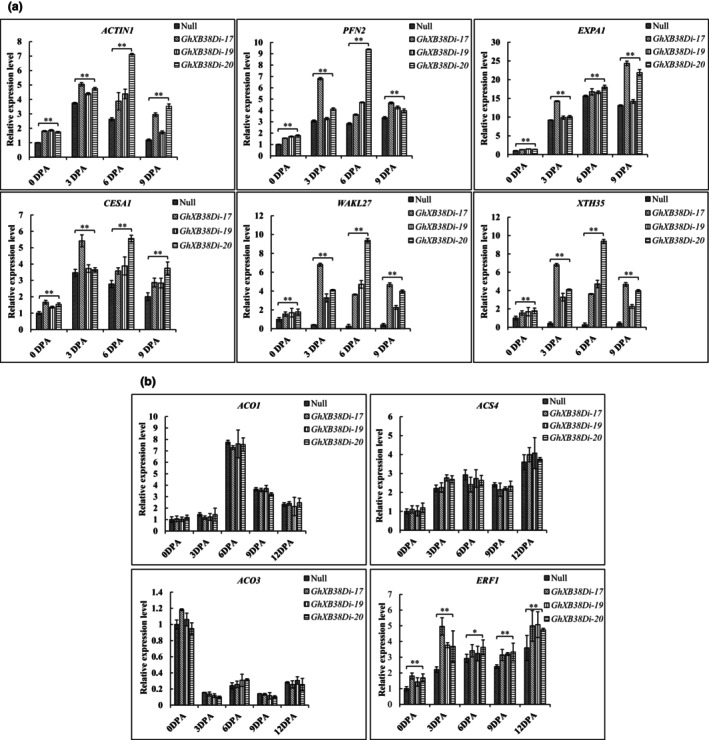
Quantitative real‐time PCR analysis of the expression of fibre‐related and ethylene signalling genes in cotton. (a) qRT‐PCR analysis of gene expression levels associated with cytoskeletal organization and cell wall construction in null plants and *GhXB38D* RNAi lines (*GhXB38Di‐17, 19* and *20*) at the stage of fibre development (0 DPA to 9 DPA). Cotton UBI was used as an internal control. Error bars represent the SD of three replicates. **P < 0.01 (based on Student's *t*‐tests). *ACTIN1*, actin 1; *PFN2*, profilin 2; *EXPA1*, expansin 1; *CESA1*, cellulose synthase gene A 1; *WAKL27*, wall‐associated kinase‐like 27; *XTH35*, xyloglucan endotransglucosylase/hydrolase 35. DPA, days post‐anthesis. (b) qRT‐PCR analysis of gene expression levels associated with ethylene synthesis and signalling (*ACO1, ACO3, ACS4* and *ERF1*) in null plants and *GhXB38D* RNAi lines (*GhXB38Di‐17, 19* and *20*) at the stage of fibre development (0 DPA to 12 DPA). Cotton UBI was used as an internal control. Error bars represent the SD of three replicates. **P < 0.01 (based on Student's *t*‐tests).

Ethylene treatment promotes fibre cell elongation in cotton by increasing the expression of genes involved in cytoskeleton construction, cell wall synthesis and cell expansion (Shi *et al*., 2006). To investigate the role of *GhXB38D* in cotton fibre elongation, we compared the transcription levels of fibre development‐related genes and ethylene signalling pathway genes during fibre elongation (from 0 to 12 DPA). In the *GhXB38Di* lines, we observed significant up‐regulation of *ACTIN1* and *PFN2*, which play direct roles in microtubule and actin cytoskeleton organization, respectively, during fibre elongation (from 0 to 9 DPA, Figure 5a). This up‐regulation is consistent with the longer fibre phenotype observed in the *GhXB38Di* lines (Figure 2c). Additionally, the transcripts of genes related to cell wall remodelling (e.g., *EXPA1*), cell wall composition (e.g., *CESA1* and *XTH35*) and signalling (e.g., *WAKL27*) were also significantly up‐regulated in the *GhXB38Di* lines (Figure 5a), which likely contributed to the observed increase in fibre strength and lint percentage (Tables 1 and 2). Furthermore, the expression of the ethylene response factor *ERF1* was up‐regulated during fibre elongation (0–12 DPA) in the *GhXB38Di* lines, indicating that the elevated ethylene content in these lines activated ethylene‐related signalling pathways. However, the expression of genes involved in ethylene synthesis (e.g., *ACO1, ACO3* and *ACS4*) did not differ significantly from that in the null plants, suggesting that GhXB38D is involved in regulating the stability of ACO1 and ACS4 proteins rather than their transcription (Figure 5b). These findings further support the role of *GhXB38D* in modulating fibre elongation by suppressing the ethylene signalling pathway.

### Elevated ethylene content in the *GhXB38D* RNAi lines contributes to enhanced fibre elongation

**Figure 6:**
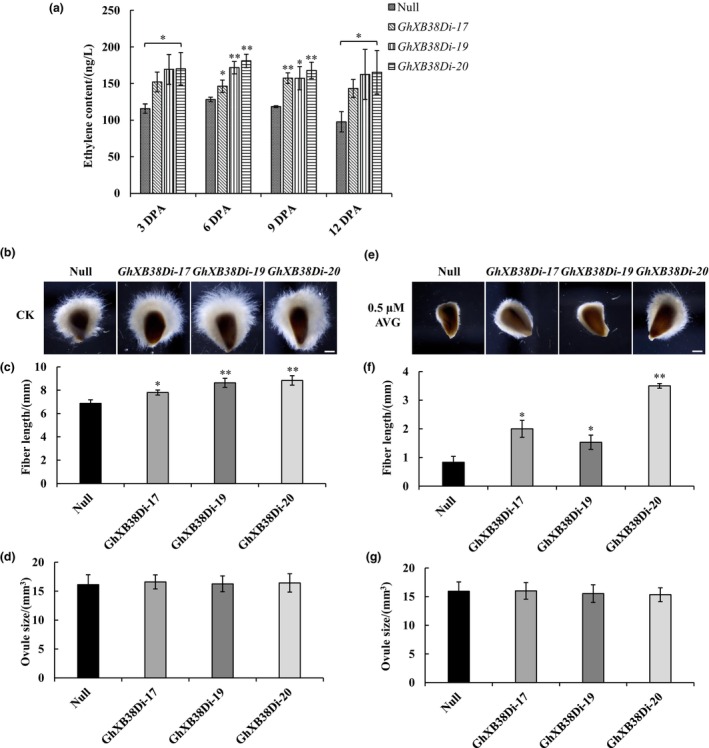
Determination of ethylene content in fibres of null plants and *GhXB38Di* lines and effect of exogenous application of AVG on elongation of ovule fibre cells. (a) ELISA determination of ethylene content in ovules of null plants and *GhXB38Di* lines (*GhXB38Di‐17, 19* and *20*) at different stages of development (from 3 DPA to 12 DPA). Error bars represent ± SE of three biological replicates (*P < 0.05; **P < 0.01, by Student's *t*‐test). (b, e) Ovules of null plants and *GhXB38Di* lines (*GhXB38Di‐17, 19* and *20*, collected at 1 DPA) were cultured for 13 days without (CK) or with 0.5 μM AVG, respectively, for phenotypic analysis. Bars = 5 mm. (c, f) Fibre lengths of null plants and *GhXB38Di* lines (*GhXB38Di‐17, 19* and *20*) measured at the end of 13 days of culture. (d, g) Ovule sizes of null plants and *GhXB38Di* lines (*GhXB38Di‐17, 19* and *20*) measured at the end of 13 days of culture. The average fibre length for each experiment was obtained from 30 ovules, and all experiments were repeated at least three times independently. Error bars represent ± SE of three biological replicates (*P < 0.05; **P < 0.01, by Student's *t*‐test).

To investigate the impact of suppressed expression of *GhXB38D* on endogenous ethylene in cotton fibres, we quantified and compared the ethylene content in the fibres of null plants and *GhXB38D* RNAi lines during the rapid fibre elongation stage (from 3 to 12 DPA). Using enzyme‐linked immunosorbent assay (ELISA), we observed increased ethylene content in *GhXB38Di* fibres compared to that of the null plants at all tested fibre development stages (Figure 6a). To further validate the effect of altered endogenous ethylene content on fibre development, we cultured the ovules of null plants and *GhXB38Di* lines *in vitro* and treated them with AVG. The results demonstrated that the fibre length of the *GhXB38Di* lines without AVG treatment was significantly longer than that of the null plants after 13 d of culture, with no significant difference in ovule size (Figure 6b–d). These findings indicate that the increase in endogenous ethylene content positively contributes to fibre elongation. After AVG treatment (0.5 μM), both *GhXB38Di* lines and null plants exhibited an inhibition of fibre elongation; however, the fibre length of *GhXB38Di* lines remained longer than that of null plants (Figure 6e–g). Western blotting analysis revealed significantly higher protein levels of GhACS4 and GhACO1 in *GhXB38Di* lines than in null plants (Figure 4d,e). Considering that AVG is a nonspecific inhibitor of ACS protein activity and hinders fibre elongation by reducing ethylene accumulation, these results further suggest that the protein levels of both GhACS4 and GhACO1 in *GhXB38Di* lines contribute to ethylene accumulation and enhanced fibre elongation.

## Discussion

### Optimal ethylene production in cotton fibres is not solely dependent on transcriptional regulation of ethylene synthesis genes

Ethylene is a gaseous phytohormone that plays a crucial role in regulating plant growth and development, fruit ripening, and cotton fibre elongation (Binder, 2020; Dubois *et al*., 2018; Xiong *et al*., 2014). Previous studies have shown that exogenous ethylene treatment or its precursor (e.g., ACC) effectively promotes fibre elongation and fruit ripening (Fang *et al*., 2017; Qin *et al*., 2007, Qin and Zhu, 2011; Shi *et al*., 2006). This led us to hypothesize that increased expression of ethylene synthesis genes would significantly elevate ethylene content in cotton fibres, thereby improving their length. However, the expression levels of ethylene synthesis genes did not exhibit a linear correlation with fibre length among different cotton cultivars (Fang *et al*., 2017; Jiang *et al*., 2022). Furthermore, the excessive accumulation of *ACO1* and *ACO3* transcripts, which are involved in ethylene synthesis, in the fibres of diploid cotton inhibit fibre elongation (Li *et al*., 2014). These findings highlight the complex relationship between the expression of ethylene synthesis genes and ethylene content in cotton fibres. Higher transcript levels of ethylene synthesis genes do not necessarily result in high ethylene content in cotton fibres. Accordingly, we observed no significant difference in *GhACS4* and *GhACO1* gene expression in the fibres of null plants and *GhXB38Di* lines (Figure 5b), despite significant differences in ethylene contents (Figure 6a). These results suggest that the ethylene content in cotton fibres is not solely regulated by the transcriptional levels of ethylene synthesis genes, such as *ACOs* and *ACS*s.

Strict regulation of the ethylene synthesis pathway is crucial to maintain optimal levels of ethylene for plant growth and development (Binder, 2020; Lyzenga and Stone, 2012; Xiong *et al*., 2014). Transgenic *Arabidopsis* plants expressing the *AtACS6* gene under the 35S promoter did not overproduce ethylene (Lyzenga *et al*., 2012). Additionally, persistently high expression of the ethylene signalling pathway genes, such as *EIN3* and *EIL1*, in *Arabidopsis*, impairs normal plant growth and development (Dolgikh *et al*., 2019; Potuschak *et al*., 2003). Furthermore, the exogenous application of high ethylene concentrations can induce senescence and dehiscence in plant tissues (Chen and Huang, 2022; Schneider *et al*., 2018). Based on these observations, we infer that the expression of ethylene synthesis genes is also regulated at the post‐translational level. In this study, we observed strong up‐regulation of *GhACS4* and *GhACO1* genes during cotton fibre development, which was synchronized with the up‐regulation of the E3 ligase gene *GhXB38D* (Figures 1b and S6). This supports the notion that maintaining moderate ethylene content for fibre development requires the post‐translational regulation of ethylene synthases.

### GhXB38D controls fibre elongating through the ubiquitination of GhACS4 and GhACO1

As rate‐limiting enzymes in the ethylene synthesis pathway, ACS and ACOs are the main targets for regulating ethylene content during plant development (Park *et al*., 2021; Wang *et al*., 2002; Yang and Hoffman, 1984). ETO1/EOL2 and XBAT32 in *Arabidopsis* have been identified as regulators of ethylene production via the ubiquitin‐dependent degradation of type‐2 and type‐3 ACS proteins, respectively (Chae *et al*., 2003; Christians *et al*., 2009; Prasad *et al*., 2010). MaXB3 modulates the stability of MaACS1 and MaACO1 to inhibit ethylene biosynthesis in banana fruits during ripening (Shan *et al*., 2020). Based on the knowledge of post‐translational modifications of ACO and ACS by XB3 or XB3‐like proteins in plants, we investigated whether cotton XB3 proteins serve as regulators of GhACO and GhACS. We identified GhXB38D as an E3 ligase that interacts with the ethylene biosynthetic enzymes GhACS4 and GhACO1 in yeast two‐hybrid assays. These interactions were further confirmed using BiFC and LCI experiments (Figure 3). Furthermore, *in vivo* and *in vitro* ubiquitination assays demonstrated that GhXB38D catalyses the degradation of GhACS4 and GhACO1 proteins (Figure 4). The increased stability of GhACS4 and GhACO1 contributed to the elevated ethylene content in the *GhXB38D* RNAi lines (Figure 6a). These findings clearly indicate that GhXB38D modulates the abundance of GhACS4 and GhACO1 proteins through ubiquitination.

To elucidate the role of *GhXB38D* in fibre elongation, we generated *GhXB38D* RNAi lines and found that the fibres of the *GhXB38Di* lines were significantly longer than those of the null plants from the early stages of fibre elongation (Figure 2c). Scanning electron microscopy (SEM) results demonstrated that the fibre cells of the *GhXB38Di* lines exhibited faster elongation starting from 3 DPA compared to the null plants (Figures 2b and S4). The *GhXB38Di* lines also showed a significant increase in the lint index and lint percentage compared with the null plants (Table 1). Moreover, the strength and elongation rate of mature fibres of *GhXB38Di* lines were increased, indicating that *GhXB38D* plays a negative regulatory role in fibre development (Table 2). In conclusion, these results demonstrate that GhXB38D negatively regulates fibre elongation and controls ethylene production in fibres by decreasing the abundance of GhACS4 and GhACO1 proteins. These findings also provide a partial explanation as to why increasing the transcript levels of *ACOs*, *ACSs* genes and ethylene‐responsive transcription factors in cotton did not significantly improve fibre length and quality (Fang *et al*., 2017; Jiang *et al*., 2022).

Multiple reports have shown that ethylene synthesis affects sucrose supply and transport during plant development. The role of *GhXB38D* in fibre elongation likely influences sucrose supply in cotton fibres. Ethylene not only activates the biosynthesis of UDP‐L‐Rha and UDP‐D‐GalA, the major components of primary cell walls (Pang *et al*., 2010), but also enhances cell elongation by up‐regulating the expression of genes related to the sucrose synthase pathway (Shi *et al*., 2006). Downstream of ethylene signalling, GhCSLD3, a cellulose synthase‐like D3 that uses UDP‐glucose as a substrate, mediates fibre cell elongation (Hu *et al*., 2018). The inhibition of *GhXB38D* in cotton regulated the expression levels of fibre cell expansion genes, such as *EXPA1* and *ACTIN1*, as well as the cellulose synthesis gene *CESA1* (Figure 5a). These findings suggest that *GhXB38D* inhibits fibre elongation, which may indirectly affect sucrose availability in elongated cotton fibres. Therefore, future studies should investigate whether *GhXB38D* affects sucrose supply while regulating ethylene production during fibre development. In summary, we have demonstrated that GhXB38D regulates ethylene synthesis by affecting the stability of GhACS4 and GhACO1 proteins through ubiquitination. Considering the critical role of ethylene synthesis catalysed by ACS and ACOs for fibre elongation and quality, the regulatory mechanism elucidated in this study holds significant importance.

## Materials and methods

### Plant materials and growth conditions

Cotton plants (*Gossypium hirsutum* cv. Jimian14, Xuzhou142 (Xu142) and Xuzhou142 fibreless mutant (*Xu142fl*)) were grown in a greenhouse at Shanghai Jiaotong University, China, from March 1 to November 30. Flowers were labelled with 0 DPA on the day of flowering, and cotton bolls were labelled and collected every 3 days after flowering. Cotton ovules were carefully collected from the bolls and immediately immersed in liquid nitrogen. Fibres were gently stripped from the ovules in liquid nitrogen and used for total RNA extraction and subsequent analysis. Roots, stems and young leaves were collected from 15‐day‐old cotton seedlings analysis of gene expression patterns. Placenta, stigma, petal, anther and carpel were also collected from post‐flowering plants and used for analysis. Samples for nucleic acid extraction were ground to a fine powder in liquid nitrogen with Tissuelyser‐192 at 55.0 HZ for 60 s and stored at −80 °C. Tobacco (*Nicotiana benthamiana*) plants were grown under long‐day conditions of 22 °C (16 h light/8 h darkness), and plants of 4–6 weeks of age were selected for Agrobacterium‐mediated transient expression assay.

### Identification and phylogenetic analysis of the *XB3* gene family in cotton

The genomic data of *G. hirsutum* cultivar TM‐1 (AD1) were obtained from the website http://mascotton.njau.edu.cn/info/1054/1118.htm, which was established by the Cotton Research Institute, Nanjing Agricultural University, while the genomic data of *G. raimondii* (D5) and *G. arboreum* (A2) were obtained from CottonGen (https://www.cottongen.org). XB3 family protein sequences of *Oryza sativa* and *Arabidopsis thaliana* were downloaded from NCBI (http://www.ncbi.nlm.nih.gov/) and used to construct a hidden Markov model (HMM), which was further used as a query to search against the predicted protein dataset comprising three cotton species using HMMER 3.1 (http://hmmer.janelia.org/) (E value ≤10^−5^). Typical ANK domain (PF12796) and RING domain (PF13920) in the hit sequences were further confirmed by searching the SMART (http://smart.embl‐heidelberg.de/smart/) and InterPro (http://www.ebi.ac.uk/interpro/) databases. Protein sequences containing both ANK and RING domains were considered as candidate XB3s. The SMART database (http://smart.embl‐heidelberg.de/) was used to remove the redundant sequences. The cotton *XB3* genes were renamed according to their homologues in the rice (*Oryza sativa*) genome (Nodzon *et al*., 2004). The plant XB3 proteins from *A. thaliana, O. sativa, G. hirsutum, G. arboretum* and *G. raimondii* were aligned using the ClustalW program (https://www.ebi.ac.uk/Tools/msa/clustalw2/). The NJ phylogenetic tree of these XB3 proteins was constructed using the MEGA7.0 program (https://www.megasoftware.net/) with 1000 bootstrap replicates.

### Gene expression analysis based on qRT‐PCR and RNA‐seq data

Total RNA was extracted from cotton samples using RNAprep Pure Plant Kit (Polysaccharides & Polyphenolics‐Rich) (Tiangen, China). One microgram of RNA was used for cDNA synthesis using PrimeScript™ RT Kit with gDNA Eraser (TaKaRa, Dalian, China). The qRT‐PCR analysis was performed according to the instructions of the SYBR premix Ex‐*Taq* kit (TaKaRa) and monitored by a Roche Light Cycler 96 real‐time PCR machine (Roche, Basel, Switzerland). The ubiquitin gene was used as an internal control (Walford *et al*., 2011). Transcriptional changes in gene expression were calculated using the comparative ΔC_T_ method (Huang *et al*., 2013). The primer sequences used for gene expression analysis are listed in Table S3.

The RNA‐seq data for cotton roots, stems, leaves and fibres (−3, 0, 1, 3, 5, 10, 15, 20 and 25 DPA) of *G. hirsutum* TM‐1 were downloaded from the NCBI database (SRA: PRJNA248163). The SRA data were converted to the fastq format using the SRA toolkit with the ‐split‐3 parameter. The reference genome sequence for *G. hirsutum* ZJU v2.1 was downloaded from the website (https://www.cottongen.org/data/download/genome_tetraploid/AD1), and the reference genome sequences for *G. hirsutum* was downloaded from https://www.cottongen.org/ and used to construct the reference genome using the Bowtie2 program (Langmead and Salzberg, 2012). The fastq data were analysed using the FastQC program, and the transcripts were mapped to the *G. hirsutum* genomes using the default parameters of the TopHat2 program (Langmead and Salzberg, 2012). The expression levels of *GhACO*s and *GhACS*s genes were calculated using Log2 (FPKM).

### *In situ* hybridization

Sense and anti‐sense probes were prepared using the DIG RNA Labeling Kit (SP6/T7) (Roche). Cotton ovules (3 DPA) were fixed with FAA, dehydrated (50%–100% ethanol) and embedded in paraffin. Hybridization assays were performed according to the enhanced sensitive ISH detection kit (AP) (BOSTER, Wuhan, China). Signals were observed with an Olympus BX51 microscope (Olympus, Tokyo, Japan).

### Generation of *GhXB38D* RNAi transgenic cotton plants

After BlastN online search (www.cottongen.org), a specific fragment of *GhXB38D* cDNA (from 70 bp to 195 bp) was inserted into the multiple cloning sites of the *pHellsgate12* plasmid to generate a *GhXB38D* silencing vector. The *pHellsgate12‐GhXB38D* RNAi plasmid was introduced into *Agrobacterium tumefaciens* strain LBA4404 for genetic transformation of cotton. Agrobacterium‐mediated transformation was performed using hypocotyl segments of *G. hirsutum* cv. Jimian14 according to the method of Luo *et al*. (2007). Transgenic positive T_0_ plants were screened on medium containing 75 mg/L kanamycin and then confirmed by PCR. Positive plants were grown in the greenhouse and self‐crossed to T_4_ lines to obtain homozygous lines for subsequent analysis. Primer sequences used for plasmid construction are listed in Table S3.

### Cotton fibre quality analysis

Healthy mature bolls (5th to 10th bolls from the top of the cotton plants) were harvested during the 2020 and 2021 growing seasons. The collected bolls were dried at 25 °C with low humidity for 1 week and used for fibre quality analysis. Fibre yield and quality index analysis were performed according to the principles reported by Sun *et al*. (2019), respectively. Lint index (fibre weight per 100 seeds in grams), seed index (seed weight per 100 seeds in grams) and lint percentage were calculated based on 50 bolls. Fibre quality parameters were calculated using a high‐capacity fibre testing system (Premier HFT 9000; Premier Evolvics Pvt. Ltd, Coimbatore, India) at the Cotton Fibre Quality Inspection and Testing Center, Ministry of Agriculture, China (Anyang City, Henan Province, China). All experiments were replicated at least three times.

### Fibre development analysis

Cotton ovules with 0 DPA and 3 DPA were isolated and directly observed by scanning electron microscope under cold vacuum (SEM; TCS SP8 STED 3X; Leica Microsystems, Wetzlar, Germany). Differences in fibre cell development between null plants and transgenic RNAi lines were analysed. Cotton fibres at different stages of fibre development (6 DPA, 9 DPA and 12 DPA) and mature fibres were collected and analysed for fibre length. The ovules were immersed in 95 °C water for 5 min to relax the fibres. Fibres were imaged under a microscope, and fibre lengths were calculated using the IMAGEJ program (http://rsb.info.nih.gov/ij/) (Xu *et al*., 2012).

### Cotton ovule culture

Flowers were harvested at 1 DPA, and ovaries were surface sterilized with 75% ethanol. Ovules were carefully dissected from the ovaries under sterile conditions and immediately floated on liquid media supplemented with or without 0.5 μM AVG in 50 mL flasks (Ashcraft, 1996; Beasley and Ting, 1973). Ovules were incubated in the dark at 30 °C without agitation. After 13 days of incubation, the fibre length of all developing ovules was measured. Cultured ovules were soaked in 95 °C water for 5 min to relax the fibres. Fibres were imaged under a microscope, and fibre lengths were calculated using the IMAGEJ program.

### Subcellular localization analysis of proteins

To investigate the subcellular localization of the GhXB38D protein and its variants, the coding region of the *GhXB38D* gene and truncated fragments encoding different domains (GhXB38D Δankyrin‐repeat/GhXB38D protein without ankyrin‐repeat domain, GhXB38D ΔRING finger/GhXB38D protein without RING finger domain, GhXB38D‐AA/GhXB38D protein with mutations in two zinc coordination residues) were cloned into the *pEarlyGate101* vector to generate *CaMV35S:: GhXB38D* or *GhXB38D* variant YFP expression cassettes. Cultures of *Agrobacterium tumefaciens* strain GV3101 carrying constructs expressing different YFP fusion proteins and P19 were mixed in a 1:1 ratio and introduced into *N. benthamiana* leaves by infiltration. After 2 days of infiltration, the subcellular localization of the proteins was analysed by confocal laser microscopy (TCP SP5; Leica Microsystems, Wetzlar, Germany). YFP was excited with a 488‐nm laser (emission, 500–535 nm).

### Yeast two‐hybrid assay

The Y2H Gateway™ adapted vectors pGBKT7 and pGADT7 were used. Full‐length cDNA and truncated fragments of the *GhXB38D* gene (GhXB38D‐N/ankyrin‐repeat domain of GhXB38D, GhXB38D‐C/RING finger domain of GhXB38D) were cloned into the pGADT7 vector to generate constructs expressing prey proteins. The full‐length *GhACS4, GhACS6* and *GhACO1~3* genes were cloned into the pGBKT7 vector to generate BD vectors. The bait and prey constructs were co‐transformed into strain AH109 by a PEG/LiAc transformation procedure (Gietz and Schiestl, 2007). After incubation at 30 °C for 3–4 days, one yeast cell was picked out and incubated in liquid synthetic dropout medium lacking Leu and Trp until OD_600_ was 0.6. Yeast cells were plated on SD‐Trp/‐Leu/‐His/‐Ade plates and incubated at 30 °C for 3–5 days to test for protein interactions. The primer sequences used to construct the plasmids are listed in Table S3.

### Bimolecular fluorescent complimentary (BiFC) analysis

For the BiFC assay, the coding sequences of *GhXB38D, GhACS4* and *GhACO1* without stop codon were cloned into *pEarlyGate201* or *pEarlyGate202* vectors, respectively, and fused to the N‐ or C‐terminal region of YFP. Expression of the target gene alone was used as a negative control. Agrobacterium cells carrying the indicated vector were resuspended in infiltration buffer containing 10 mM MgCl_2_, 10 mM MES and 150 μM acetosyringone to a final concentration of OD_600_ = 0.5. Agrobacterium cultures were then mixed 1:1 and infiltrated into 2‐week‐old *N. benthamiana* leaves. Fluorescence of the recombinant YFP protein was observed on tobacco leaves after 2 days of infiltration using a TCS SP5 confocal laser scanning microscope.

### Firefly luciferase complementation imaging (LCI) assay

Fusion proteins of GhXB38D, GhACS4 and GhACO1 with the N‐ or C‐terminal region of YFP are transiently expressed in *N. benthamiana* (Grefen *et al*., 2010). The empty cLUC and nLUC vectors alone were used as controls. These constructs were transformed into *A. tumefaciens* (strain GV3101) carrying the helper plasmid pSoup‐P19. Agrobacterium cells containing cLUC‐GhXB38D, GhACS4‐nLUC or GhACO1‐nLUC were suspended in infiltration buffer and then mixed in a 1:1 volume ratio. Infection of 2‐week‐old tobacco leaves with the Agrobacterium mixture. The infiltrated leaves were harvested and sprayed with 0.5 mM D‐fluorescein and potassium salt. Tobacco plants were kept in the dark for 6 min to quench fluorescence. A low‐light cooled CCD imaging device was used to capture the LUC image as described (He *et al*., 2004).

### *In vitro* ubiquitination assay

*In vitro* ubiquitination assays were performed as previously described (Zhao *et al*., 2013). Recombinant proteins His‐GhACS4/GhACO1 and MBP‐GhXB38D were purified from *E. coli* using 50 ng purified E1 UBA2, 50 ng purified E2 UBC10, 1 μg Ub (Boston Biochem), 1 μg MBP‐GhXB38D and 0.5 μg His‐GhACS4/GhACO1 were incubated in 30 μL ubiquitination reaction buffer (50 mM Tris‐Cl pH 7.5, 20 mM MgCl_2_, 5 mM ATP, 5 mM MgCl_2_, 1 mM DTT) at 28 °C for 2 h. Proteins were separated by SDS‐PAGE, and ubiquitinated His‐GhACS4/GhACO1 was detected with anti‐His antibody. Self‐ubiquitination of MBP‐GhXB38D was detected using anti‐MBP antibody.

### *In vivo* proteasome degradation assay

The full‐length cDNA of *GhXB38D* without the stop codon and *GhXB38D*‐AA were cloned into the *pEarleyGate*105 vector to generate mCherry‐*GhXB38D*/*GhXB38D*‐AA. The coding regions of *GhACS4* and *GhACO1* were then inserted into the Gateway binary vector *pGWB505* to generate GFP‐*GhACS4*/*GhACO1*. Equal volumes of *A. tumefaciens* strain GV3101 containing different combinations of each construct were mixed and co‐infiltrated into *N. benthamiana* leaves.

Leaves were harvested and ground to a fine powder in liquid nitrogen. For MG132 treatment, leaves were infiltrated with 10 mM MG132 or distilled water and incubated for 12 h before harvesting. Cell‐free proteasome degradation assays were performed as previously described (García *et al*., 2014). Briefly, total protein extracts from 1 g of fresh leaves were prepared by beading in 250 μL degradation/DNase digestion buffer and incubated for 30 min at room temperature to a final reaction volume of 50 μL. Protein concentrations were determined using Bradford reagent (Bio‐Rad Laboratories). Reactions were terminated by boiling in SDS sample buffer, followed by SDS‐PAGE and immunoblot analysis using anti‐GFP antibody (Abcam).

### Preparation of anti‐GhACS4 and anti‐GhACO1 polyclonal antibodies

The full‐length coding sequences of *GhACO1* and *GhACS4* were cloned into pET28a (+) and transformed into *Escherichia coli* strain BL21 (DE3). Affinity purification of the recombinant protein was performed using Ni‐NTA His binding resin (Novagen). His fusion proteins were separated by sodium dodecyl sulfate‐polyacrylamide gel electrophoresis. Bands of interest were excised and used as antigens for antibody production. Antibodies were produced in rabbits by YINGJI Biotechnology Company.

### Western blot analysis

Analysis of GhACO1 and GhACS4 protein levels in null plants and *GhXB38Di* lines. Total proteins from ovules and fibres of 6 DPA and 9 DPA of null plants and *GhXB38Di* lines were extracted with extraction buffer [125 mM Tris–HCl, (pH 8.0), 375 mM NaCl, 2.5 mM EDTA, 1% SDS, and 1% β‐mercaptoethanol], and then proteins were separated in 10% SDS‐PAGE gels and transferred to the PVDF nylon membrane. The proteins on the membrane were analysed with anti‐GhACO1/GhACS4 antibodies (AB clonal, Wuhan, China) using the chemifluorescence hybridization kit.

### Statistical analysis

One‐way analysis of variance (ANOVA) method was used to analyse all data in this study. Error bars represent the SD from three biological replicates of three parallel experiments. Asterisks indicate significant differences between controls and different treatments (*P* < 0.05 or 0.01, Student's *t*‐test).

### Accession numbers

Sequence data from this article can be found in the GenBank data libraries (https://www.ncbi.nlm.nih.gov) under the following accession numbers: GhXB38D (XP_016671180), ACO1 (DQ116442), ACO2 (DQ116443), ACO3 (DQ116444), ACS4 (JF508505), ACS6 (DQ122174), ERF1 (GD254630), UBI7 (DQ116441), ACTIN1 (AY305723), PFN2 (GU237487), EXPA1 (AY189969), CESA1 (U58283), WAKL27 (XM_016853487) and XTH35 (EF546795).

## Conflict of interest

The authors declare no conflicts of interest.

## Author contributions

Zuo K.J. and Wang J. designed and conducted the experiments. Song Q.W., Gao W.T., Sun W.J. and Du C.H. performed the experiments. Zuo K.J. and Song Q.W. wrote and revised the paper.

## Supporting information


**Figure S1** The *GhXB38D* gene encodes a typical RING E3 ligase with an ankyrin‐repeat domain and a C3HC4‐type RING finger domain. (a) A phylogenetic tree was constructed with MEGA 7.0 using the neighbour‐joining (NJ) method with 1000 bootstrap replicates based on a multiple alignment of XB3 proteins from *Gossypium hirsutum*, *Gossypium raimondii*, *Gossypium barbadense*, *Arabidopsis thaliana* and *Oryza sativa*. The XB3 proteins are grouped into three distinct clades. (b) Schematic representation of the GhXB38D protein domain. (c) GhXB38D aligned with *Arabidopsis* XB3 proteins. Black line indicates ankyrin‐repeat domain and black dashed line indicates RING finger domain. Red boxes indicate two zinc‐binding residues.
**Figure S2** RNA in situ hybridization shows the distribution of the *GhXB38D* signal in cotton ovules at 3 days post‐anthesis (DPA). f, fibre; osc, outer seed coat; isc, inner seed coat.
**Figure S3** PCR characterization of *GhXB38D* RNAi transgenic cotton plants. (a) Schematic of the expression cassette of the *GhXB38D* RNAi construct used in cotton transformation. (b) PCR characterization of *GhXB38D* RNAi cotton plants. M, DL‐2000 molecular marker; PC, positive control; NC, negative control; 1–20, transgenic *GhXB38D* RNAi cotton plants.
**Figure S4** Fibre lengths of the ovule surfaces of null plants and *GhXB38Di* lines at 3 DPA. The average length of cotton fibres was calculated using 30 seeds from null plants and the *GhXB38Di* lines in the scanning electron microscope. Error bars represent the SD of three replicates. **P < 0.01 (based on Student's *t*‐tests).
**Figure S5** Vegetative phenotypes of null plants and *GhXB38Di* cotton lines. (a) Plant phenotypes of null plants and *GhXB38Di* cotton lines. (b) Growth parameters of null plants and *GhXB38Di* cotton lines.
**Figure S6** Quantitative real‐time PCR analysis of the expression of ethylene biosynthesis‐related genes in cotton fibres. qRT‐PCR analysis of the expression levels of *ACO1, ACO2, ACO3, ACS4* and *ACS7* genes in cotton fibres (from 0 to 12 DPA). Cotton UBI was used as an internal control. Error bars represent ± SE of three biological replicates. Error bars indicate the standard error of the independent biological replicates (*P < 0.05; **P < 0.01, by Student's *t*‐test).
**Figure S7** BiFC assays show increased fluorescence intensity of protein interactions in the presence of MG132 treatment and GhXB38D‐AA. (a) Interaction of GhXB38D (or GhXB38D‐AA) with GhACO1 and GhACS4 was detected by BiFC assays after MG132 treatment in tobacco leaves. GhXB38D‐AA indicates mutations in two zinc‐binding residues of the GhXB38D protein. Bars = 50 μM. (b) Analysis of fluorescence intensity of protein interactions in tobacco leaves. Mean grey values and integrated density were measured using ImageJ.
**Table S1** Basic characteristics of XB3 family genes in the genomes of Gossypium hirsutum, Gossypium raimondii, Gossypium barbadense, Arabidopsis thaliana and Oryza sativa.
**Table S2** Expression profiles of ACS and ACO gene families in G. hirsutum.
**Table S3** Primer sequences used in the study.
