# Supplementary material for: GhXB38D represses cotton fibre elongation through ubiquitination of ethylene biosynthesis enzymes GhACS4 and GhACO1
Source: Plant Biotechnol J. 2023 Aug 19;21(11):2374–88. doi: 10.1111/pbi.14138 (PMC10579717; doi:10.1111/pbi.14138)
Supplement: Supplementary file 1 — Figure S1 The GhXB38D gene encodes a typical RING E3 ligase with an ankyrin‐repeat domain and a C3HC4‐type RING finger domain. (a) A phylogenetic tree was constructed with MEGA 7.0 using the neighbour‐joining (NJ) method with 1000 bootstrap replicates based on a multiple alignment of XB3 proteins from Gossypium hirsutum, Gossypium raimondii, Gossypium barbadense, Arabidopsis thaliana and Oryza sativa. The XB3 proteins are grouped into three distinct clades. (b) Schematic representation of the GhXB38D protein domain. (c) GhXB38D aligned with Arabidopsis XB3 proteins. Black line indicates ankyrin‐repeat domain and black dashed line indicates RING finger domain. Red boxes indicate two zinc‐binding residues. Figure S2 RNA in situ hybridization shows the distribution of the GhXB38D signal in cotton ovules at 3 days post‐anthesis (DPA). f, fibre; osc, outer seed coat; isc, inner seed coat. Figure S3 PCR characterization of GhXB38D RNAi transgenic cotton plants. (a) Schematic of the expression cassette of the GhXB38D RNAi construct used in cotton transformation. (b) PCR characterization of GhXB38D RNAi cotton plants. M, DL‐2000 molecular marker; PC, positive control; NC, negative control; 1–20, transgenic GhXB38D RNAi cotton plants. Figure S4 Fibre lengths of the ovule surfaces of null plants and GhXB38Di lines at 3 DPA. The average length of cotton fibres was calculated using 30 seeds from null plants and the GhXB38Di lines in the scanning electron microscope. Error bars represent the SD of three replicates. **P < 0.01 (based on Student's t‐tests). Figure S5 Vegetative phenotypes of null plants and GhXB38Di cotton lines. (a) Plant phenotypes of null plants and GhXB38Di cotton lines. (b) Growth parameters of null plants and GhXB38Di cotton lines. Figure S6 Quantitative real‐time PCR analysis of the expression of ethylene biosynthesis‐related genes in cotton fibres. qRT‐PCR analysis of the expression levels of ACO1, ACO2, ACO3, ACS4 and ACS7 genes in cotton fibres (from 0 t [file PBI-21-2374-s001.zip › supporting information/Supplemetary Figures.pdf]

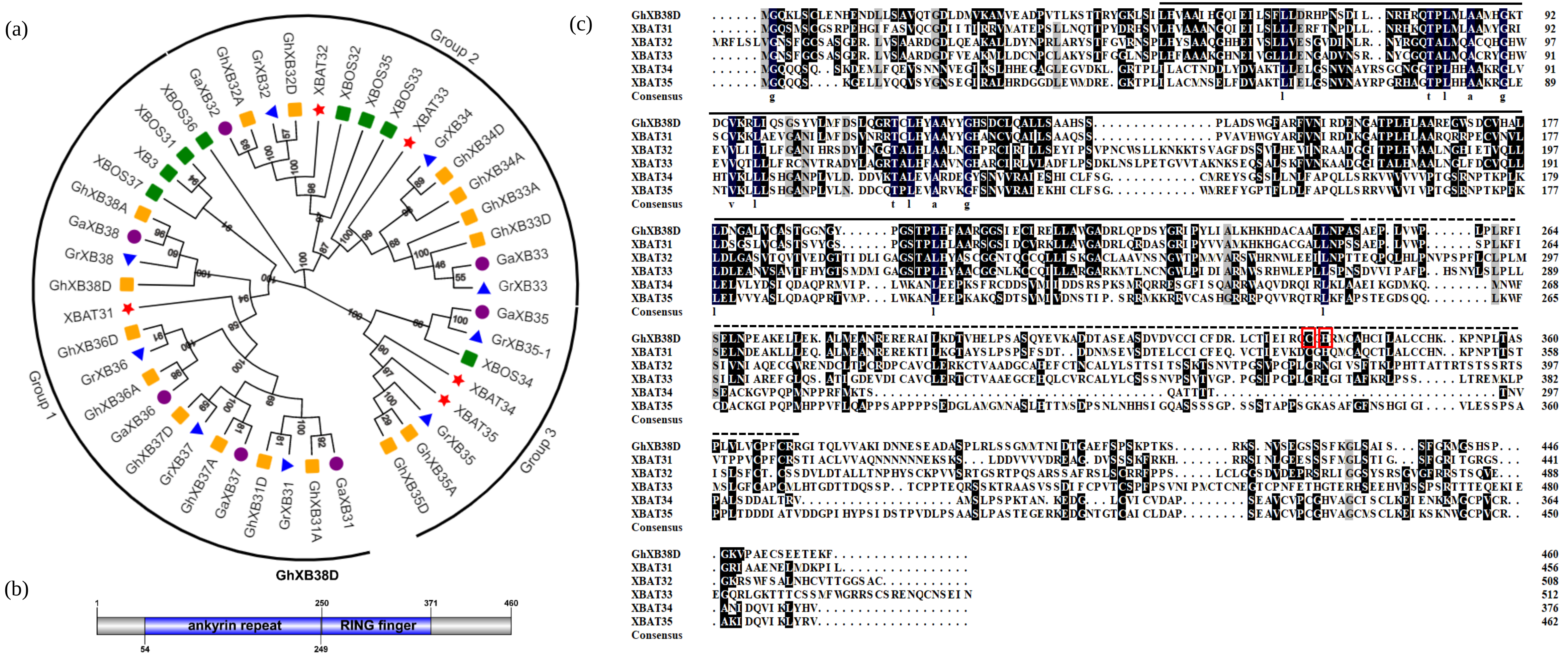

Figure S1. The *GhXB38D* gene encodes a typical RING E3 ligase with an ankyrin repeat domain and a C3HC4-type RING finger domain.

(a) A phylogenetic tree was constructed with MEGA 7.0 using the neighbor-joining (NJ) method with 1000 bootstrap replicates based on a multiple alignment of XB3 proteins from *Gossypium hirsutum*, *Gossypium raimondii*, *Gossypium barbadense*, *Arabidopsis thaliana*, and *Oryza sativa*. The XB3 proteins are grouped into three distinct clades.
(b) Schematic representation of the GhXB38D protein domain.
(c) GhXB38D aligned with *Arabidopsis* XB3 proteins. Black line indicates ankyrin repeat domain, black dashed line indicates RING finger domain. Red boxes indicate two zinc-binding residues.

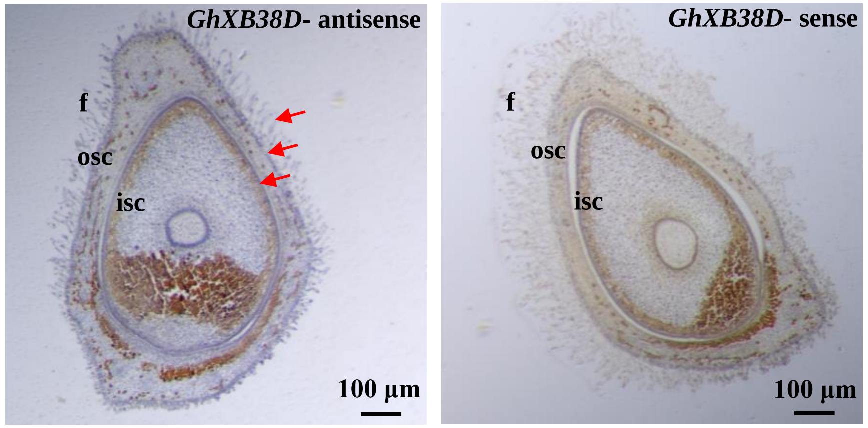

Figure S2. RNA in situ hybridization shows the distribution of the *GhXB38D* signal in cotton ovules at 3 days post anthesis (DPA). f, fiber; osc, outer seed coat; isc, inner seed coat.

(a)

(b)

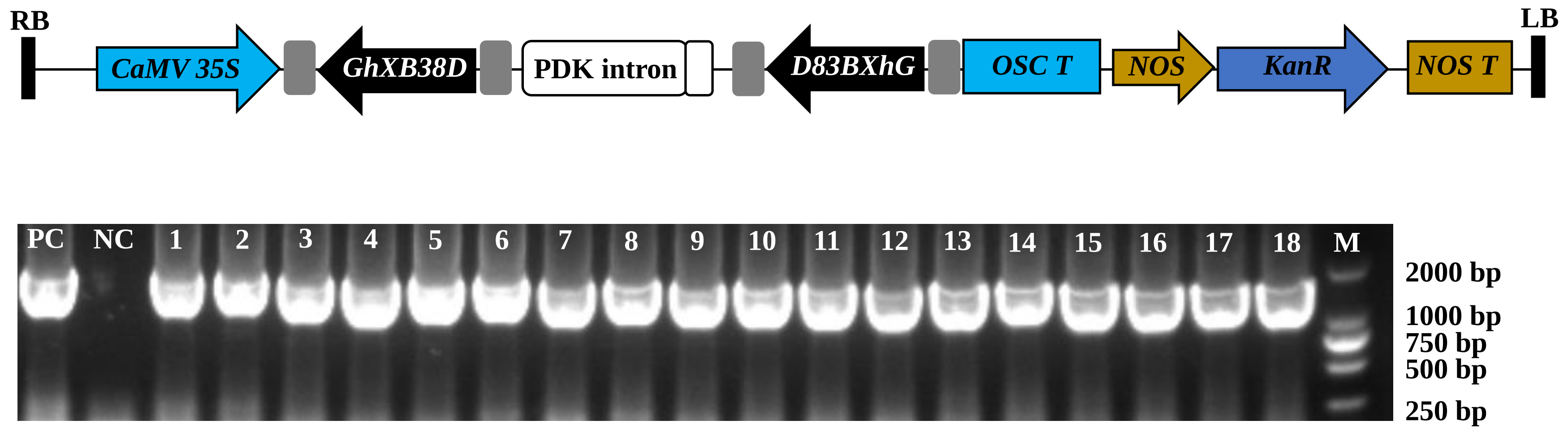

Figure S3. PCR characterization of *GhXB38D* RNAi transgenic cotton plants.

(a) Schematic of the expression cassette of the *GhXB38D* RNAi construct used in cotton transformation..
(b) PCR characterization of *GhXB38D* RNAi cotton plants. M: DL-2000 molecular marker; PC: positive control; NC: negative control; 1-20: transgenic *GhXB38D* RNAi cotton plants.

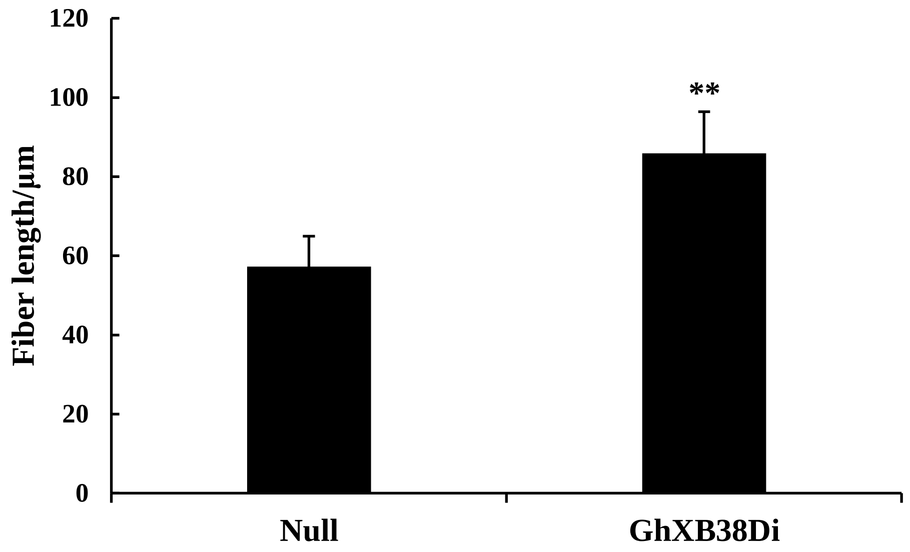

Figure S4. Fiber lengths of the ovule surfaces of null plants and *GhXB38Di* lines at 3DPA.

The average length of cotton fibers was calculated using 30 seeds from null plants and the *GhXB38Di* lines in the scanning electron microscope. Error bars represent the SD of three replicates. **, $P < 0.01$ (based on Student's *t*-tests).

(a)

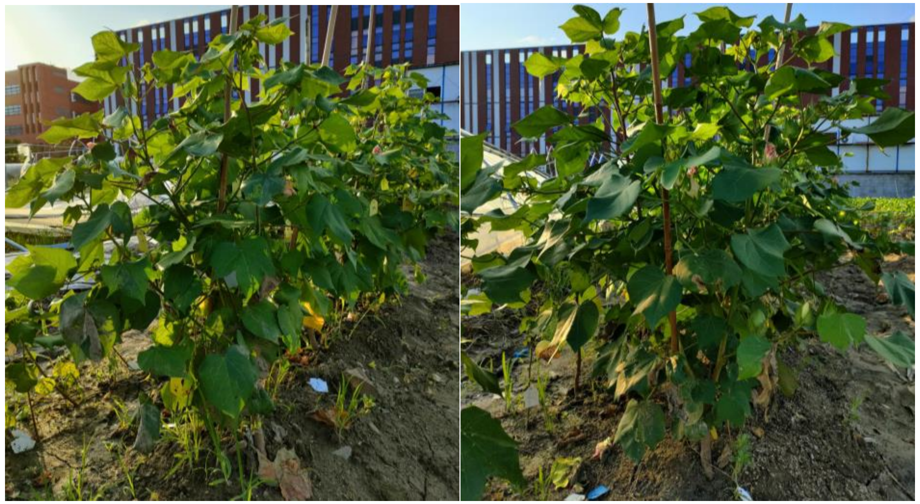

Null ***GhXB38Di***

(b)

		Flowering time	Plant height/(cm)	Boll number
2020	Null	2020.7.20	126.6±3.40	63±4.90
	GhXB38Di-17	2020.7.16	127.67±3.09	65.33±7.13
	GhXB38Di-19	2020.7.21	127.33±5.25	62±5.35
	GhXB38Di-20	2020.7.17	132±3.27	58.33±3.30
2021	Null	2021.7.21	126.67±6.80	58±4.55
	GhXB38Di-17	2021.7.22	136±4.32	57.67±4.19
	GhXB38Di-19	2021.7.18	133±6.68	61.67±4.92
	GhXB38Di-20	2020.7.21	132±4.90	60.67±6.94

Figure S5. Vegetative phenotypes of null plants and *GhXB38Di* cotton lines.

(a) Plant phenotypes of null plants and *GhXB38Di* cotton lines.

(b) Growth parameters of null plants and *GhXB38Di* cotton lines.

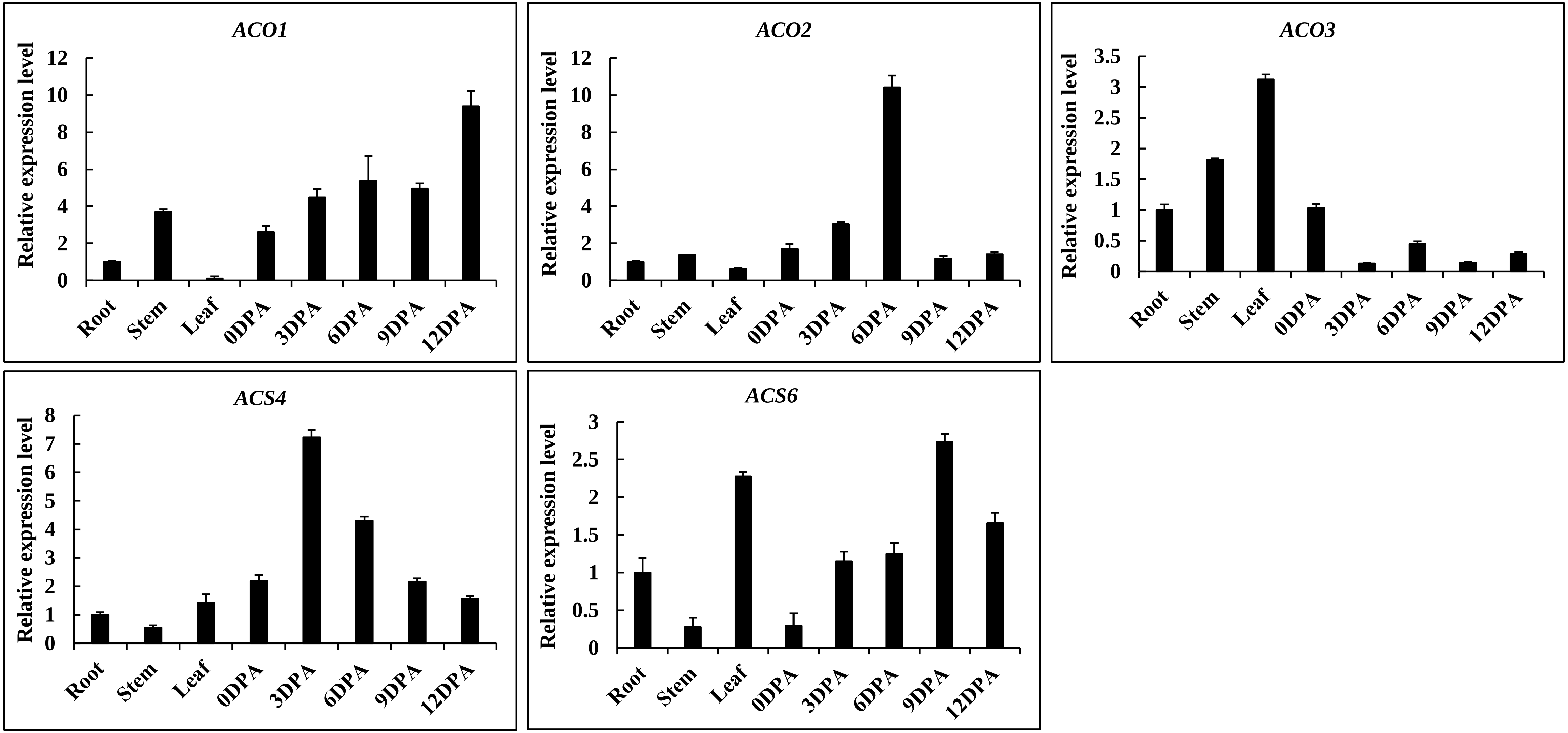

Figure S6. Quantitative real-time PCR analysis of the expression of ethylene biosynthesis-related genes in cotton fibers.

qRT-PCR analysis of the expression levels of *ACO1*, *ACO2*, *ACO3*, *ACS4* and *ACS7* genes in cotton fibers (from 0 to 12 DPA). Cotton UBI was used as an internal control. Error bars represent ± SE of three biological replicates. Error bars indicate the standard error of the independent biological replicates (*$P < 0.05$; **$P < 0.01$, by Student's *t*-test).

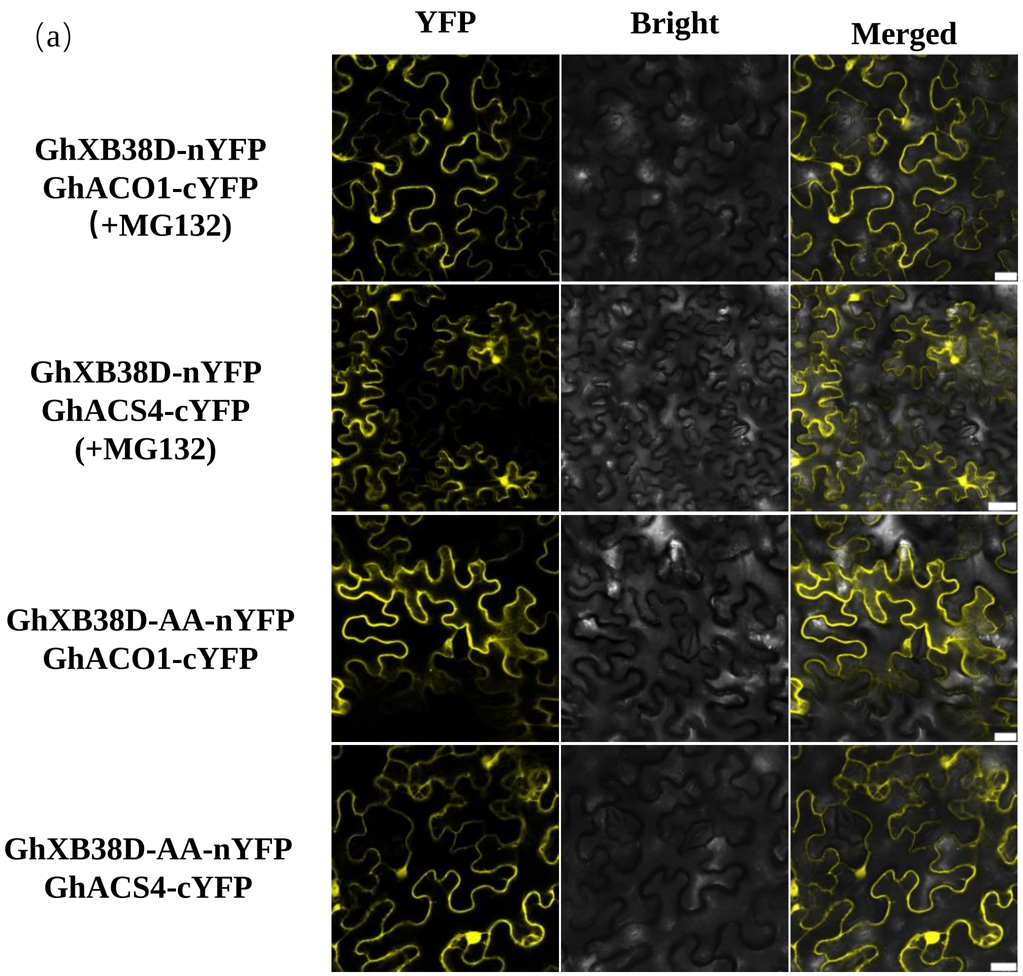
(a)
YFP
Bright
Merged
GhXB38D-nYFP
GhACO1-cYFP
(+MG132)
GhXB38D-nYFP
GhACS4-cYFP
(+MG132)
GhXB38D-AA-nYFP
GhACO1-cYFP
GhXB38D-AA-nYFP
GhACS4-cYFP

(b)

	GhXB38D-nYFP/ GhACO1-cYFP (+MG132)	GhXB38D-nYFP/ GhACS4-cYFP (+MG132)	GhXB38D-AA-nYFP/ GhACO1-cYFP	GhXB38D-AA-nYFP/ GhACS4-cYFP	GhXB38D-nYFP/ GhACO1-cYFP	GhXB38D-nYFP/ GhACS4-cYFP
Mean grey value	12.736	18.18	16.945	15.937	7.663	8.530
Integrated density	221914	316774	297494	277689	135555	149761

Figure S7. BiFC assays show increased fluorescence intensity of protein interactions in the presence of MG132 treatment and GhXB38D-AA.

(a) Interaction of GhXB38D (or GhXB38D-AA) with GhACO1 and GhACS4 was detected by BiFC assays after MG132 treatment in tobacco leaves. GhXB38D-AA indicates mutations in two zinc-binding residues of the GhXB38D protein. Bars = 50 μM.

(b) Analysis of fluorescence intensity of protein interactions in tobacco leaves. Mean gray values and integrated density were measured using ImageJ.
